# Congenital Hypothyroidism Due To Maternal Radioactive Iodine Exposure During Pregnancy

**DOI:** 10.4274/jcrpe.553

**Published:** 2012-06-09

**Authors:** Selim Kurtoğlu, Mustafa Ali Akın, Ghaniya Daar, Leyla Akın, Şeyma Memur, Levent Korkmaz, Osman Baştuğ, Selcan Yılmaz

**Affiliations:** 1 Erciyes University Faculty of Medicine, Department of Pediatrics, Division of Neonatology, Kayseri, Turkey; 2 Nevşehir Government Hospital, Department of Pediatrics Endocrinolog, Nevşehir, Turkey; 3 Nevşehir Government Hospital, Department of Pediatrics, Nevşehir, Turkey; +90 352 437 49 37seymamail@yahoo.com

**Keywords:** Pregnancy, Hyperthyroidism, radioactive iodine, fetal hypothyroidism

## Abstract

Radioactive iodine (RAI) is used effectively in the treatment of hyperthyroidism and thyroid cancer, but it is contraindicated during pregnancy. RAI treatment during pregnancy can lead to fetal hypothyroidism, mental retardation and increased malignancy risk in the infant. Pregnancy tests must be performed before treatment in all women of reproductive age. However, at times, RAI is being used before ruling out pregnancy.

We herein present a male newborn infant with congenital hypothyroidism whose mother was given a three-week course of methimazole therapy for her multiple hyperactive nodules and subsequently received 20 mCi RAI during the 12th week of her pregnancy. The patient was referred to our neonatology unit at age two weeks when his thyrotropin (TSH) level was reported to be high in the neonatal screening test. Physical examination was normal. Laboratory investigations revealed hypothyroidism (free triiodothyronine 1.55 pg/mL, free thyroxine 2.9 pg/mL, TSH 452 mU/L, thyroglobulin 20.1 ng/mL). The thyroid gland could not be visualized by ultrasonography. L-thyroxine treatment was initiated.

**Conflict of interest:**None declared.

## INTRODUCTION

Radioiodine (I131) is a convenient, inexpensive, safe and effective treatment for hyperthyroidism and thyroid malignancy in children and adults (1,2). The use of I131 is absolutely contraindicated during pregnancy principally because of the risk of damaging the fetal thyroid gland and thus leading to hypothyroidism or cretinism (1,2,3,4,5,6). Neonatal hyperthyrotropinemia is observed if the applieddose is below 10 mCi (7). Also, hypothyroidism and hypoparathyroidism can concomitantly occur as a result of maternal radioactive iodine (RAI) treatment (8). Here, we report a young female patient who delivered a hypothyroid baby after she was given RAI treatment accidentally, being unaware that she was at the 12th week of her pregnancy at the time of therapy.

## CASE REPORT

During the screening programme for congenital hypothyroidism, a fifteen-day old male infant was found twice to have a thyrotropin (TSH) level exceeding 600 mU/L. The patient was referred to the neonatology unit of Erciyes University Faculty of Medicine. Medical history revealed that the mother had received methimazole therapy for 3 weeks due to multiple hyperactive nodules and that this was followed by RAI treatment (20 mCi). Subsequent to this treatment, the mother was detected to be at the 12th week of her pregnancy. It was reported that the mother remained euthyroid after the treatment and this was given as the reason why fetal thyroid functions were not measured. The baby was born spontaneously via the vaginal route at the end of a 42-week pregnancy. At birth, body weight was 3840 g, length was 52 cm and head circumference was 36.5 cm. On the 15^th^ postnatal day, the infant was 55 cm in length, weight was 4720 g, and head circumference was 37 cm. His anterior fontanelle dimensions were 4x6 cm and those for the posterior fontanelle were 0.5x0.5 cm. Otherwise, physical examination was normal. The umbilical cord was still undetached.

A plain knee X-ray showed findings consistent with a37-week gestation. Thyroid volume was measured as 0.1 mL(normal= 0.8) by ultrasonography. Free triiodothyronine was 1.55 pg/mL (normal= 2.99-6.66), free thyroxine 2.9 pg/mL (normal= 6.6-23.7), thyrotropin (TSH) 452 mU/L (normal= 0.70-18.10), thyroglobulin level 20.1 ng/mL (normal= 91), urine iodine level was 3 μg/dL (normal= 10-20 μg/dL). TSH receptor antibody level of the maternal serum was 2.4 U/L (normal= 0-10 U/L). The baby was started on L-thyroxine therapy at a dose of 15 μg/kg and is now being followed by our team.

## DISCUSSION

Thyrotoxicosis in pregnancy can be treated using antithyroid drugs as the first choice. Rarely, thyroidectomy can also be an option, but RAI is contraindicated (9). RAI is not used in known pregnancies, but its use in undetected pregnancies is rarely reported (1,6). RAI given to a pregnant woman crosses the placenta rapidly and reaches the fetus (10). After the 12th week of gestation, the fetal thyroid gland starts uptaking and storing iodine (11). Fetal serum RAI level reaches 75% of mother’s serum level and RAI can persist in the fetal thyroid gland for approximately 70-75 days (11,12). All fetal tissues, and especially the thyroid tissue, are 2-3 times more sensitive to radioactivity as compared to adult tissues (13). RAI concentrated in the thyroid gland causes ablation (11,13,14). As was also the case in our patient, RAI uptake increases and causes more severe injury in iodine-deficient fetuses (15). Therefore, probably due to its total destruction by RAI, the thyroid gland could not be detected by ultrasonography in our patient.

RAI can create a lethal effect on the embryo and can negatively affect brain development both directly by its radioactive effect and indirectly by creating hypothyroidism (13,14). Besides hypothyroidism, a fetus exposed to RAI can have mental retardation, malformations, as well as an increased cancer risk in the later years of its life (16). In some reported cases, hypoechoic thyroid nodules were detected and resolved with thyroxine therapy (17).

To prevent RAI exposure during pregnancy, it is crucial to perform a proper pregnancy test in hyperthyroid female patients and not just rely on medical history. For this purpose, the American College of Radiology has prepared a guideline listing 4 different clinical situations that eliminate the possibility of pregnancy (18). These are: 1- A negative result in a pregnancy test performed within the past 72 hours, 2- A history of hysterectomy, 3- A state of menopause for at least two years, 4- A premenarcheal child aged 10 years or younger. Also, one should keep in mind that pregnancy tests relying on urine or serum hCG levels are not totally reliable in the first 8-10 postconceptional days, since implantation may not yet have been completed during this period (19).

In conclusion, for women in reproductive ages who require RAI treatment, the importance of performing a pregnancy test 3 days prior to the treatment needs to be emphasized. If a woman who has received RAI therapy is later detected to be pregnant, fetal thyroid function tests should be undertaken and prenatal treatment should be initiated (6).
